# The ubiquitin ligase c-CBL is expressed in undifferentiated marmoset monkey pluripotent stem cells but is not a general stem cell marker

**DOI:** 10.5194/pb-4-231-2017

**Published:** 2017-11-20

**Authors:** Ignacio Rodriguez-Polo, Maike Nielsen, Katharina Debowski, Rüdiger Behr

**Affiliations:** 1 Platform Degenerative Diseases, German Primate Center (DPZ), Leibniz Institute for Primate Research, Kellnerweg 4, 37077 Göttingen, Germany; 2 DZHK, German Center for Cardiovascular Research, Partner Site Göttingen, Göttingen, Germany; a present address: STEMCELL Technologies Germany GmbH, Stolberger Str. 200, 50933 Cologne, Germany; * These authors contributed equally to this work

## Abstract

The protein c-CBL is a ubiquitin ligase. It catalyzes the last step of the transfer of ubiquitin to target proteins. Upon completion of polyubiquitination, the target proteins are degraded. Clinically, it is important that c-CBL is mutated in a subset of patients who develop myeloid malignancies, which are diseases of the hematopoietic stem or progenitor cells. c-CBL has also been shown to be expressed by human spermatogonia. The whole spermatogonial cell population possesses a subset that comprises also the spermatogonial stem cells. Based on these findings we hypothesized that c-CBL might be a general stem cell marker. To test this, we first validated the antibody using marmoset bone marrow and adult testis. In both tissues, the expected staining pattern was observed. Western blot analysis revealed only one band of the expected size. Then, we examined the expression of c-CBL in marmoset monkey embryonic stem (ES) cells, induced pluripotent stem (iPS) cells and adult stem cells. We found that c-CBL is strongly expressed in undifferentiated marmoset iPS cells and ES cells. However, adult stem cells in the gut and the stomach did not express c-CBL, indicating that c-CBL is not a general stem cell marker. In summary, c-CBL is strongly expressed in pluripotent stem cells of the marmoset monkey as well as in selected adult stem cell types. Future studies will define the function of c-CBL in pluripotent stem cells.

## 1 Introduction

It is well known that premeiotic germ cells and induced pluripotent stem (iPS) cells share the expression of many pluripotency-associated factors in both rodents and primates. For instance, we have recently shown that marmoset monkey primordial germ cells (PGCs), which are the embryonic precursors of the gametes, express the key pluripotency factors OCT4A (POU5F1) and NANOG as well as SALL4 and LIN28 (Aeckerle et al., 2015), which are all also expressed by pluripotent stem cells. The two latter ones are also expressed in different populations of adult spermatogonia (Aeckerle et al., 2012; Eildermann et al., 2012). Spermatogonia are the premeiotic germ cells in the adult testis and also comprise the spermatogonial stem cell population. The close relationship between premeiotic germ cells and pluripotent stem cells such as embryonic stem (ES) cells is not only based on the remarkable overlap in protein expression, but also on the fact that (at least mouse) PGCs can be stably converted in culture to pluripotent germ-line-derived stem cells (Matsui et al., 1992; Resnick et al., 1992; Guan et al., 2006; Kanatsu-Shinohara et al., 2004) and mouse pluripotent stem cells can be converted to germ cells (Saitou and Miyauchi, 2016). Hence, although germ cells are in vivo physiologically unipotent, there is a considerable similarity between premeiotic germ cells and pluripotent stem cells with regard to their gene expression signature and, under experimental conditions, their developmental potential.

The c-CBL protein (named after Casitas B-lineage Lymphoma) is an E3 ubiquitin ligase first discovered in 1989 (Langdon et al., 1989). E3 ubiquitin ligases catalyze the transfer of ubiquitin from the E2 ligase to the target protein, which may then be degraded or targeted to other cellular processes. It is well known that E3 ligases play important roles in cell cycle control (Teixeira and Reed, 2013), and c-CBL has been shown to ubiquitinate protein tyrosine kinases, thereby leading to degradation of these receptors (Mohapatra et al., 2013). Moreover, combined gene deletion of *c-CBL* and *CBL-b* in mice showed embryonic lethality at embryonic day 10, suggesting an important role of the protein family members in (stem) cell development and function. Furthermore, a role of c-CBL and CBL-b for T-cell function was documented (Naramura et al., 2002). c-CBL also plays an important clinical role. Myeloid malignancies originate from hematopoietic stem or progenitor cells of the myeloid lineage and mutations in the *c-CBL* gene have been found in many patients with this type of blood cell malignancies (Murati et al., 2012; Katzav and Schmitz, 2015; Lv et al., 2017). Using a combined microarray and immunohistochemistry approach, von Kopylow and colleagues (von Kopylow et al., 2010) further demonstrated that c-CBL is expressed in the human testis specifically by spermatogonia. The published data on the role c-CBL in the development of myeloid malignancies and its expression by testicular spermatogonia may suggest that c-CBL plays a general role in stem cells. Based on this, we hypothesize that pluripotent stem cells express c-CBL and that c-CBL may be a general adult stem cell marker. Both hypotheses were tested in this study.

## 2 Material and methods

### 2.1 Marmoset monkey stem cell lines

The generation and culture of the pluripotent stem cell lines used in this study were reported previously (Mueller et al., 2009; Debowski et al., 2015, 2016).

### 2.2 Immunofluorescence

Marmoset stem cells grown on mouse embryonic feeder (MEF) cells were washed twice with PBS (phosphate-buffered saline), fixed in PBS, 2 % (w/v) PFA and 0.02 % (v/v) TritonX-100 (30 min, RT, room temperature), and washed twice with PBS and 5 % (w/v) BSA (bovine serum albumin). Incubation with NANOG (Cell Signaling Technology 1E6C4 mAb, monoclonal antibody) and c-CBL (Sigma HPA027956, generated against human c-CBL) antibodies, diluted in PBS and 5 % (w/v) BSA, was conducted overnight at 4 ${}^{\circ }$C. Cells were washed twice with PBS and 5 % (w/v) BSA, and Alexa488-coupled secondary donkey anti-rabbit antibody (Life Technologies) diluted 1:900 together with Alexa569-coupled secondary goat anti-mouse antibody (Life Technologies) diluted 1:300 in PBS and 5 % (w/v) BSA were applied for 30 min at RT. Cells were incubated with DAPI in PBS, washed twice with PBS and finally coated with Citifluor mounting medium (Citifluor Ltd.). Images were taken with a Zeiss Observer Z1 (Zeiss).

### 2.3 Non-directed differentiation of iPSCs

Marmoset iPSCs were transferred from mouse embryonic feeder cells to a feeder-free Geltrex attachment matrix (Geltrex^®^ hESC-Qualified, Ready-To-Use, Reduced Growth Factor Basement Membrane Matrix; Thermo Fisher), cultured for 2 days with Essential 8 (E8) medium (Thermo Fisher) and supplemented with pro-survival compound (5 µM, Calbiochem) for the first 24 h. To induce differentiation, the medium was changed to M10 medium (DMEM, Gibco; 10 % (v/v) Fetal Bovine Serum, Gibco; 1 % (v/v) Penicillin–Streptomycin, Gibco; 0.25 µg mL${}^{-1}$ Amphotericin B, Sigma; 1 % (v/v) MEM Non-Essential Amino Acids Solution, Gibco; 2 mM GlutaMAX, Gibco). Immunofluorescence of the resulting colonies was performed in the same way as specified above (Sect. 2.2).

### 2.4 Immunohistochemistry

Marmoset tissues were taken from the DPZ Platform Degenerative Diseases tissue archive. All tissues were obtained in full accordance with the German Animal Protection Act (Deutsches Tierschutzgesetz). After the sacrifice of the animals by experienced veterinarians, the tissues were immediately prepared and fixed in Bouin's solution, further processed according to routine histological techniques and eventually embedded in paraffin for immunohistochemical analysis. Sections (5 µm) were cut and placed on adherent slides. Paraffin sections were dewaxed and rehydrated. Antigen retrieval was carried out with the 10 mM Na–citrate buffer pH 6.0 for 10 min in a high-power microwave. Sections were washed for 5 min in 0.05 M Tris wash buffer. Then blocking of peroxidase was performed for 15 min with the Peroxidase Blocking Reagent (DAKO). After washing again with washing buffer, immunostaining was performed with a c-CBL antibody (Sigma HPA027956) at a 1:100 dilution in washing buffer plus 5 % BSA (w/v). Sections were incubated with the primary antibody at 4 ${}^{\circ }$C overnight. The primary antibody was detected using the DAKO HRP (horseradish peroxidase) kit (no. K8024) or the DAKO AP (alkaline phosphatase) kit (no. K5361). Antibody binding was visualized with DAB (HRP) or permanent red (AP). Mayer's hematoxylin staining was used as counterstain. Negative controls were performed by omitting primary antibodies and by using corresponding nonspecific IgG controls at the same protein concentration as the primary antibody. Images were captured with the Aperio CS2 histological tissue scanner from Leica using the program Aperio ScanScope.

### 2.5 c-CBL transcript abundance analysis

Transcriptome analyses were previously performed for marmoset fibroblasts and iPS cells (Debowski et al., 2015; GEO series accession number GSE64966) as well as marmoset ES cells (Debowski et al., 2016; GEO series accession number GSE70897). c-CBL expression data were extracted from these data sets and analyzed using the Student's t test.

### 2.6 Protein extraction and western blot

Protein from liver and cultured cells (iPSCs, ESCs and skin fibroblasts) was isolated using the Qproteome Nuclear Protein Kit from Qiagen. The protein concentration of the different samples was estimated by the Bradford assay. For western blot analysis, 20 µg of the protein lysate (including 1× DTT and 1× loading buffer) were loaded into a NuPAGE Novex 4–12 % Bis-Tris gel to separate proteins. Proteins were then transferred to a nitrocellulose membrane. The membrane was washed in PBS-T (1× PBS with 0.1 % Tween-20) and blocked for 30 min in 5 % skim milk, 0.1 % normal goat serum and PBS-T. Primary antibody incubation was performed for 1 h at room temperature. c-CBL antibody (Sigma HPA027956) was diluted 1:300 in 5 % skim milk in PBS-T. After washing in PBS-T, membranes were incubated with a secondary HRP-conjugated antibody (goat–anti-mouse–HRP from R&D Systems, no. HAF007). As size reference MagicMark XP Western Protein Standard from Thermo Fisher was used. Signals were visualized using the chemoluminescence ECL Western Blotting Analysis System (GE Healthcare, RPN2108) and the Intas ChemoCam western blot imaging system equipped with the ChemoStar professional software (Intas Science Imaging Instruments, Göttingen, Germany).

### 2.7 RNA isolation and RT-PCR for the detection of c-CBL isoforms

RNA isolation for reverse transcription polymerase chain reaction (RT-PCR) was performed using the NucleoSpin^®^ TriPrep kit from MACHEREY-NAGEL (Düren, Germany). Reverse transcription was performed using the Omniscript^®^ reverse transcription kit from Qiagen (Hilden, Germany) with oligo(dT) primers. Two RT-PCRs were performed. The first RT-PCR was performed on RNA from four different ES cell lines (Debowski et al., 2016), four iPS cell lines (Debowski et al., 2015), skin fibroblasts (second passage fibroblasts of a female marmoset monkey, cultured in M10 medium), testis, heart, muscle, liver and bone marrow (all of an adult male marmoset monkey) using the KOD Hot Start DNA Polymerase (Merck Millipore, Germany) and run for 30 cycles. c-CBL primers (annealing temperature 61 ${}^{\circ }$C) were Fw (forward primer) 5${}^{\prime }$-CTGATTGGGCTCATGAAGGAC-3${}^{\prime }$ and Rv (reverse primer) 5${}^{\prime }$-GCTTTGGGTTCTGACACAACCG-3${}^{\prime }$. Actin primers (annealing temperature 60 ${}^{\circ }$C) producing a band of 562 bp were used previously (Debowski et al., 2015). Oligonucleotides were purchased from Sigma. The second RT-PCR was performed using one sample of ESCs, iPSCs, testis and cultured fibroblast. The conditions and primers for the CBL amplification were the same as the ones described above. For the actin amplification, however, we used Fw 5${}^{\prime }$-TGGATGATGATATCGCTGCAC-3${}^{\prime }$ and Rv 5${}^{\prime }$-GAGTCCTTCTGACCCATGCC-3${}^{\prime }$, generating an amplicon of 154 bp (annealing temperature 66,4 ${}^{\circ }$C). The RT-PCR designed to specifically detect the large CBL isoform (2; Fig. 8b) was performed using the following primers: Fw 5${}^{\prime }$-CTTCCAGCCGCACCACCACCA-3${}^{\prime }$ and Rv 5${}^{\prime }$-GCTTTGGGTTCTGACACAACC-3${}^{\prime }$ (annealing temperature 61 ${}^{\circ }$C). PCR products were separated along with a low molecular weight DNA marker for better a resolution of the DNA fragments in 3.5 % agarose gel.

### 2.8 Protein sequence comparison

All c-CBL protein sequences were retrieved from the ensemble database (https://www.ensembl.org/Callithrix_jacchus/Gene/Summary?db=core;g=ENSCJAG00000014015;r=11:16861320-16951218). The longest c-CBL form of the respective species was used. For pairwise protein sequence alignments the EMBOSS Needle online tool was used (http://www.ebi.ac.uk/Tools/psa/emboss_needle/). For the generation of the *c-CBL* gene tree, MEGA 7.0 software was used, applying the UPGMA method.

## 3 Results

### 3.1 Validation of the c-CBL antibody for the marmoset monkey

**Figure 1:**
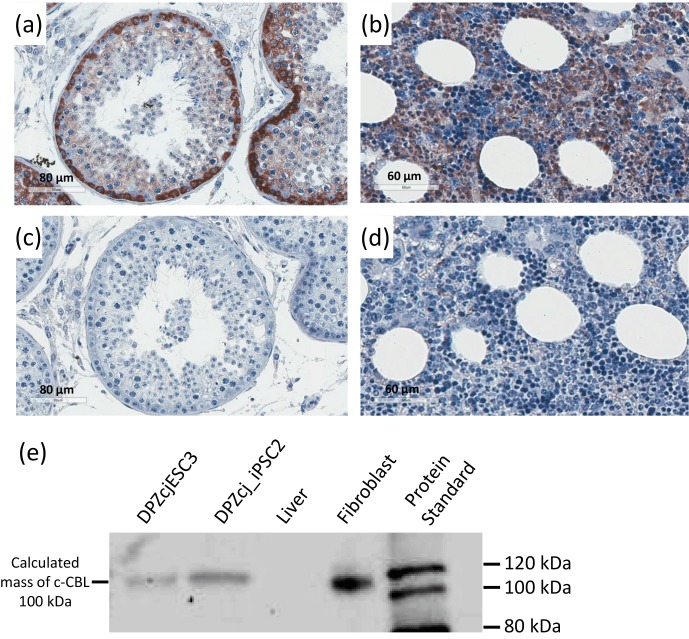
Validation of the c-CBL antibody for the marmoset. **(a)** Immunohistochemical localization of c-CBL in the marmoset testis. **(b)** Immunohistochemical localization of c-CBL in marmoset bone marrow. **(c)** and **(d)** show IgG controls corresponding to **(a)** and **(b)**. **(e)** Non-quantitative western blot of protein lysates from ESCs (DPZcjESC3), iPSCs (DPZcj_iPSC2), liver and skin fibroblasts to ensure the detection of a protein of the expected size also in marmoset samples. Only one band of the apparent size of ∼ 110 kDa is visible in the stem cell samples and fibroblasts. The calculated size of c-CBL is 100.67 kDa. The data indicate specific binding of the antibody also to marmoset c-CBL.

c-CBL is involved in the development of myeloid malignancies (Katzav and Schmitz, 2015), which originate from the bone marrow. Furthermore, c-CBL has been shown to be expressed by human spermatogonia (von Kopylow et al., 2010). Therefore, we first tested whether the c-CBL antibody used in this study produced plausible staining patterns on the respective marmoset tissues serving as positive controls. As shown in Fig. 1, both testicular spermatogonia, i.e., the germ cells located in the periphery of the cross section of the seminiferous tubules, and bone marrow showed clear cytoplasmic staining (Fig. 1a, b). In the testis, somatic cells, including almost all interstitial cells, peritubular cells and Sertoli cells, were not stained. Meiotic spermatocytes showed weak and postmeiotic spermatids only very faint staining, if at all. The corresponding IgG controls showed no staining (Fig. 1c, d). In addition, we performed (non-quantitative) western blot analysis in order to verify that the antibody specifically detects a protein of the expected size. We used marmoset ES cells, iPS cells, skin fibroblasts and liver tissue. Liver tissue was used as a biological negative control, since immunohistochemical staining of the liver showed only very few signals of lower intensity (data not shown). The calculated molecular weight of c-CBL is 100.67 kDa. We detected one specific band with an apparent molecular weight of ∼ 110 kDa in ES cells, iPS cells and fibroblasts, while liver tissue showed no band of this size (Fig. 1e). These data indicate that the antibody used in this study, which was generated against human c-CBL, also reliably detects marmoset c-CBL protein. This finding is further corroborated by the high sequence identity between marmoset and human c-CBL proteins (see below).

### 3.2 The *c-CBL* transcript is abundant in marmoset iPS and ES cells

**Figure 2:**
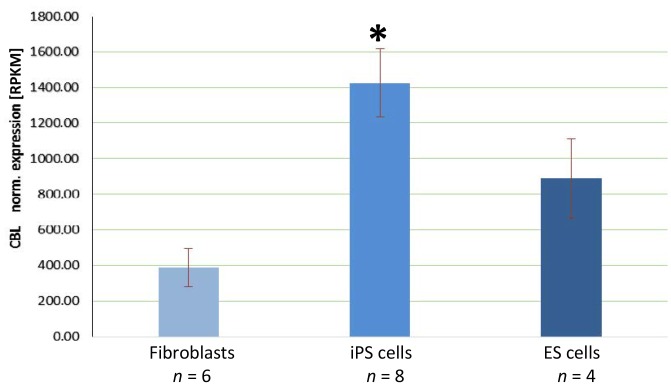
Normalized *c-CBL* transcript abundance in marmoset fibroblasts, iPS cells and ES cells. The expression levels are expressed as Reads Per Kilobase per Million mapped reads (RPKM). ${}^{*}p=$ 8.9 × 10${}^{-8}$ compared to fibroblasts.

The expression of c-CBL in the positive controls, i.e., bone marrow and spermatogonia, is associated with stem cells/stemness. However, so far the expression of c-CBL in pluripotent stem cells has not been tested. Therefore, we first analyzed *c-CBL* expression on the transcript level using our published transcriptome data (Debowski et al., 2015, 2016). *c-CBL* transcripts are highly abundant in marmoset iPS cells with a normalized mean value of 1425.63 Reads Per Kilobase per Million mapped reads (RPKM; Fig. 2) and the individual *c-CBL* RPKM values of each cell line were always in the top 10 % of the abundances of all detected transcripts. In contrast to these high values in iPS cells, marmoset fibroblasts, serving as the starting cell population for the generation of the iPS cells, have an approximately 4-fold lower *c-CBL* transcript abundance (388.33 RPKM; p= 8.9 × 10${}^{-8}$, Fig. 2). These data show that *c-CBL* transcripts are significantly more abundant in marmoset iPS cells than in fibroblasts. As reference, we also analyzed the *c-CBL* transcript abundance in marmoset ES cells and found an average expression of 889.75 RPKM. Compared to the fibroblasts, this is a significantly increased value (p= 0.023). However, compared to the iPS cells, this value is significantly lower (p= 0.015). In summary, *c-CBL* transcripts are strongly expressed in marmoset iPS and ES cells and belong to the group of highly abundant transcripts in these cells.

**Figure 3:**
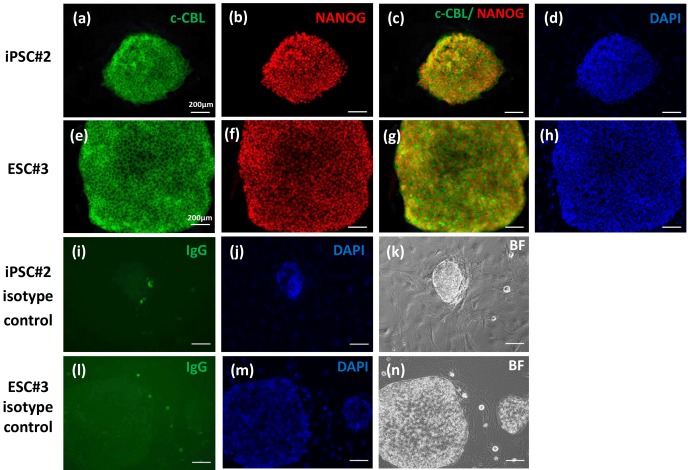
Immunofluorescence analysis of c-CBL expression in comparison with NANOG expression in cultured marmoset iPS and ES cells. The stem cells were cultured on mouse embryonic feeder (MEF) cells. The upper panels **(a–d)** show the iPS cell line 2, while **(e)**–**(h)** show the ES cell line 3. **(i)**–**(k)** and **(l)**–**(n)** show the respective IgG controls and the bright field (BF) images of the control colonies.

In order to confirm c-CBL expression in pluripotent stem cells on the protein level, we performed immunofluorescence analysis (Fig. 3) in addition to the western blot analysis shown in Fig. 1e. When we stained iPS cells or ES cell colonies cultured on mouse embryonic feeder cells for CBL; compact cell colonies with the typical morphology of undifferentiated stem cells (e.g., see bright field image Fig. 3n) were robustly and intensely stained, while the MEF cells were also not stained, further substantiating the specificity of the c-CBL staining in marmoset stem cells. (Fig. 3a, e). In order to corroborate the expression of c-CBL in undifferentiated pluripotent stem cells, NANOG, a marker of undifferentiated stem cells was also demonstrated in the same individual colonies as CBL (Fig. 3b, c, f, g). These data show that at the RNA and protein level c-CBL is robustly expressed in pluripotent marmoset stem cells.

### 3.3 c-CBL is down-regulated during differentiation in marmoset iPS cells

**Figure 4:**
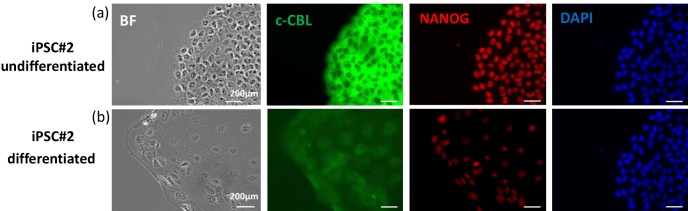
Immunofluorescence analysis of c-CBL in differentiated iPS cells in feeder-free conditions. The marmoset iPS cells were differentiated in plates coated with Geltrex matrix in M10 medium for 2 days **(b)** or kept undifferentiated in Essential 8 medium. The differentiation-associated changes in morphology (bright field image in the lower panel) are associated with decreased c-CBL and NANOG signal intensities compared to the control condition **(a)**.

In order to test whether c-CBL is down-regulated during the differentiation of the stem cells, we transferred iPS cells and ES cells from feeder-supported cultures to feeder-free conditions. After an adaptation period of 2 days in Essential 8 medium, this pluripotent stem cell medium was replaced by M10 medium for 2 days in order to induce differentiation. As control, some of the cells were kept undifferentiated in E8. Then the cells were double stained for c-CBL and NANOG (Fig. 4). The cells exposed to M10 medium show a clear decrease in the expression of CBL and also in NANOG (Fig. 4, bottom panel), while the non-differentiated control cells (Fig. 4, upper panel) still exhibited strong fluorescence signals for c-CBL and NANOG. These results indicate that c-CBL is down-regulated during the differentiation of marmoset iPSCs (Fig. 4) and ESCs (data not shown).

### 3.4 c-CBL is not a general stem cell marker

**Figure 5:**
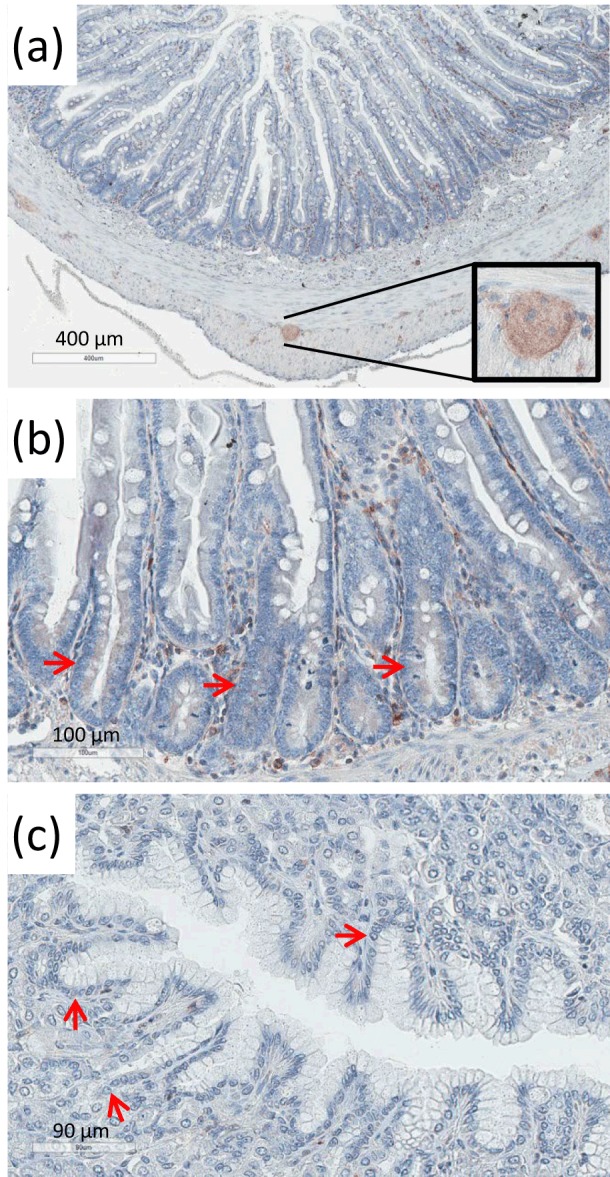
Immunohistochemical detection of c-CBL in the marmoset gut and stomach. **(a)** Low-power magnification showing a cross section of the gut. While the cells in the basal part of the crypts are not stained, the ganglia between the two peripheral muscle layers were stained (see inset). **(b)** Higher magnification of the central part of **(a)**. The sites where the stem cells are located in the crypt are labeled with red arrows. No c-CBL staining was visible. **(c)** Section of the adult stomach. No staining was visible in the glands at the sites where the endodermal epithelial stem cells are located (red arrows).

Knowing that c-CBL is involved in three different stem cell systems (bone marrow, spermatogonia, pluripotent stem cells), we wondered whether c-CBL might be a general stem cell marker. In order to test this hypothesis, we stained adult marmoset tissues that contain highly active stem cell populations, including the gut (Fig. 5a, b; Takashima et al., 2013) and the stomach (Fig. 5c; Kim and Shivdasani, 2016). However, no staining was observed in these tissues at the histological sites where the stem cells are located (Fig. 5), while SOX9 as an endodermal stem cell marker was detectable (data not shown). In contrast, ganglia of the gut (Fig. 5a, inset) and the stomach (not shown) as well as immune cells in the intestinal mesenchyme showed clear staining, proving that the staining procedure technically worked properly. The Human Protein Atlas also indicates the absence of c-CBL expression in epithelial cells of the human stomach, duodenum and small intestine (https://www.proteinatlas.org/ENSG00000110395-CBL/tissue). We also stained nonhuman primate (NHP) skin samples in order to test whether epidermal stem cells may be c-CBL positive. However, only very few insulated epidermal cells were faintly stained, thus not allowing robust conclusions. In summary, from these immunohistochemical stainings we conclude that c-CBL is not a general marker of adult marmoset stem cells.

**Table 1:** Amino acid (AA) sequence comparison of c-CBL of different primate and non-primate species. The number below each species indicates the number of AA residues of the protein. The upmost number in each box gives the percent identity and the middle number the percent similarity. The lowest number gives the number of gaps in each comparison.

Versus	Human	Chimpanzee	Rhesus	Baboon	Marmoset	Pig	Dog	Rat	Mouse
	906 AAs	906 AAs	907 AAs	907 AAs	909 AAs	914 AAs	908 AAs	914 AAs	913 AAs
Human	100 %	100 %	98.9 %	98.9 %	98.1 %	93.2 %	93.1 %	92.0 %	92.6 %
	100 %	100 %	99.2 %	99.2 %	98.7 %	95.6 %	95.6 %	94.4 %	94.8 %
	0	0	1	1	3	12	22	12	13
Marmoset	98.1 %	98.1 %	98.1 %	98.1 %	100 %	92.4 %	92.4 %	91.6 %	92.0 %
	98.7 %	98.7 %	98.8 %	98.8 %	100 %	95.1 %	95.0 %	94.3 %	94.6 %
	3	3	2	2	0	15	25	13	14

### 3.5 The c-CBL amino acid sequence is highly conserved in primates

**Figure 6:**
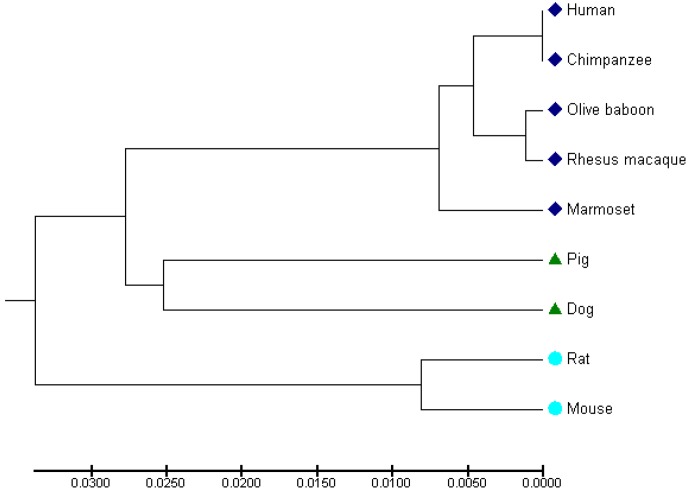
Dendrogram illustrating the evolutionary relation of c-CBL across different mammalian species. The graphic representation was generated using MEGA 7 software (UPGMA method) for c-CBL sequences from human (ENST00000264033), baboon (ENSPANG00000018962), rhesus macaque (ENSMMUG00000004761), marmoset monkey (ENSCJAG00000014015), pig (ENSSSCG00000015115), dog (ENSCAFG00000012100), rat (ENSRNOT00000067902.3) and mouse (ENSMUST00000206720.1).

High conservation of the amino acid (AA) sequence of a given protein in different species (“orthologous proteins”) usually indicates conserved roles of the protein. In order to get a first indication that our findings in the marmoset may also have relevance for other primate species, we compared the marmoset c-CBL protein sequence with the respective sequences of other primates as well as non-primate mammals (Table 1 and Fig. 6). The marmoset c-CBL protein consists of 909 amino acids and the human c-CBL protein of 906 AAs. The respective AA sequences are highly conserved, with 98.1 % identity and 98.7 % similarity (Table 1). Compared to the human, the chimpanzee (*Pan troglodytes*) c-CBL is 100 % conserved and respective values for both the rhesus monkey (*Macaca mulatta)* and the olive baboon (*Papio anubis)* are 98.9 (identity) and 98.8 % (similarity). An alignment of the NHP sequences with the human sequence revealed only one (rhesus and baboon) and three (marmoset) gaps (insertion or loss of an amino acid so that no unbroken alignment at these specific positions was possible), respectively. When the marmoset c-CBL sequence was used as reference for the comparison with the other primate sequences, the identity was in all cases 98.1 % and the similarity was either 98.7 % (human and chimpanzee) or 98.8 % (rhesus and baboon) (Table 1). The comparison of the human and the marmoset sequences with non-primate species (pig, dog, rat, mouse) revealed identities ranging from 91.6 to 93.2 % and similarities from 94.3 to 95.6 % (Table 1). Furthermore, the number of gaps in the sequence alignments between human and the non-primate species ranged from 12 to 22 and the respective range for the alignments between marmoset and the non-primate species was 13–25 (Table 1, Fig. 6). These data indicate that c-CBL is highly conserved within the group of primates and that there are considerable sequence deviations between the primate c-CBL proteins and the corresponding non-primate proteins. These findings are illustrated by the dendrogram shown in Fig. 6.

### 3.6 c-CBL isoforms in the marmoset monkey

**Figure 7:**
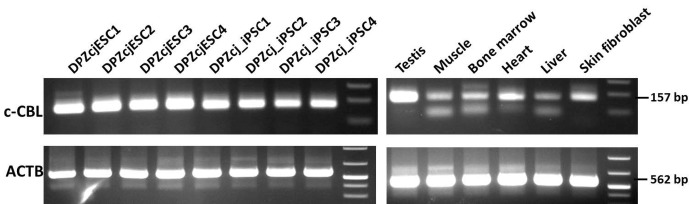
RT-PCR isoform analysis of c-CBL transcripts. The cell lines, testis and cultured fibroblasts exclusively express the larger isoform represented by the 157 bp band, which includes the 30 nt (nucleotide) variant sequence in exon 1 encoding a poly histidine stretch. The lower band appearing in muscle, bone marrow and liver may indicate the presence of the isoform lacking the 30 nt stretch. Beta-actin served as positive control. The gel of the RT-PCR negative controls (-RT, minus reverse transcriptase control) was completely blank (not shown).

**Figure 8:**
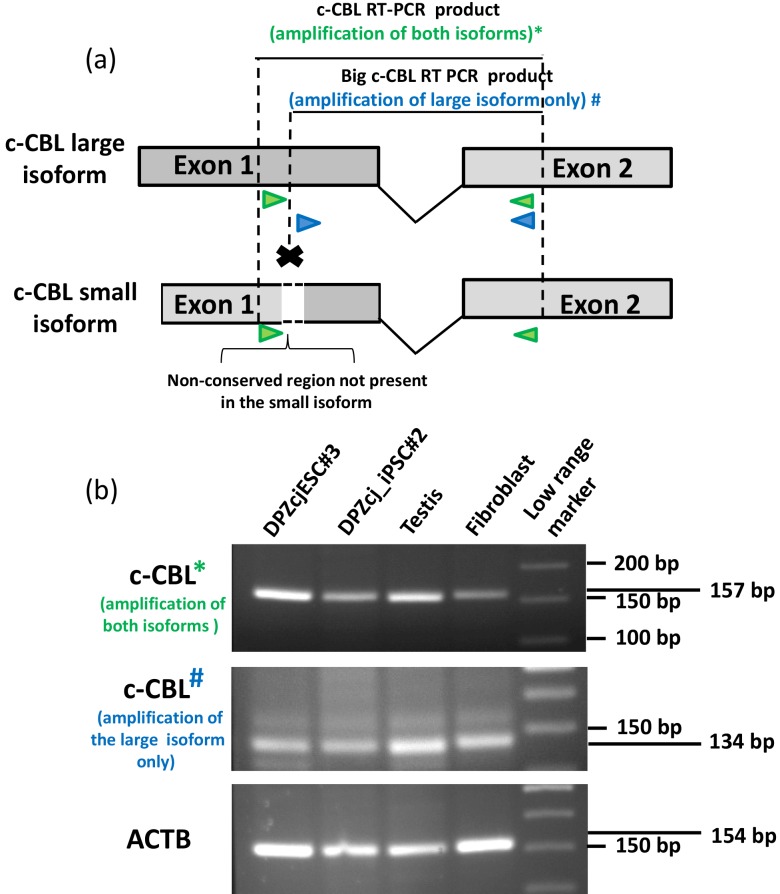
RT-PCR isoform confirmation of *c-CBL* transcripts. **(a)** Scheme of the two different RT-PCR approaches performed. The green arrows indicate the position of the primers used to amplify both isoforms simultaneously (RT-PCR 1). The blue arrows indicate the primers that allow for the specific amplification of the large isoform (RT-PCR 2), while the short isoform cannot be amplified. **(b)** High resolution agarose gel (3.5 %) electrophoresis showing the results of RT-PCR 1, RT-PCR 2 and beta actin as control. The upper gel clearly shows for RT-PCR 1 that the *c-CBL* band is slightly larger (157 bp) than the 150 bp fragment of the marker. In contrast to RT-PCR 1, RT-PCR 2 shows a band corresponding of the expected size of 134bp indicating also the expression of the larger band.

The marmoset c-CBL gene consists of 16 exons (http://www.ensembl.org/Callithrix_jacchus/Transcript/Exons?db=core;g=ENSCJAG00000014015;r=11:16861320-16951218;t=ENSCJAT00000027260), and all exons contribute to the open reading frame. However, the ensemble database indicates the existence of two marmoset c-CBL isoforms, which differ in the presence/absence of a proline residue followed by stretch of nine histidines encoded by the first exon. While the transcript with the ensemble identifier ENSCJAT00000027260.1 encodes these 10 AAs, the transcript ENSCJAT00000027254.1 lacks them. Since no data were available about the expression of the two different isoforms, we performed RT-PCR with primers flanking the region of interest in the first exon. The forward primer binds upstream in the first exon, while the reverse primer binds to a sequence in exon 2. The intron between exon 1 and 2 has 13 755 base pairs, preventing amplification of the respective genomic DNA fragment. The expected RT-PCR product indicating the longer form has a size of 157 bp, while the shorter form has a size of 127 bp. We tested c-CBL isoform expression by RT-PCR in four ES cell lines and four iPS cell lines, and we selected primary tissues (Fig. 7). All stem cell lines showed a single band of 157 bp size, suggesting the exclusive presence of the isoform containing the 30 bp encoding the stretch of the 10 AAs. In testis and cultured skin fibroblasts, this larger band was also detectable. In contrast, in muscle, bone marrow, heart and liver, smaller bands were also detectable in addition to the large band. In order to test whether these smaller bands may represent the 127 bp fragment, corresponding to the transcript ENSCJAT00000027254.1 (Fig. 7), we performed an additional RT-PCR with a forward primer binding to the variable stretch of 30 bases present only in the large isoform. This approach allowed the exclusive amplification of the large c-CBL isoform (Fig. 8). Finally, we sequenced the bands obtained in the RT-PCR and confirmed the identity of the expected amplicons. These findings shown in Figs. 7 and 8 suggest that some tissues may express both transcripts at considerable levels, while the stem cell lines, fibroblasts and the testis express only the full-length transcript at detectable levels.

## 4 Discussion

*c-CBL* plays an important role in myeloid cells since mutations in this gene result in myeloid malignancies (Murati et al., 2012; Katzav and Schmitz, 2015; Lv et al., 2017). The role of c-CBL in human spermatogonia has not been investigated in detail so far since, to our knowledge, the fertility status and testicular histology of the patients carrying c-CBL mutations has not been analyzed. Furthermore, the primate, including human, testis is experimentally almost inaccessible. Therefore mechanistic and mutational analyses of the role of c-CBL in primate spermatogonia remains almost impossible, and the role of c-CBL in primate spermatogonia therefore remains elusive. However, as shortly outlined in the introduction, pluripotent stem cells and premeiotic germ cells share some characteristics including the expression of several genes involved in the regulation of pluripotency like OCT4A and NANOG (Aeckerle et al., 2015). Here we show that adult marmoset spermatogonia also share c-CBL expression with marmoset ES cells and iPS cells. Importantly, in contrast to primate spermatogonia, which currently cannot be expanded and cultured over long periods of time, and which are therefore hardly experimentally accessible, pluripotent stem cells can be cultured over long periods of time, expanded, and manipulated by siRNA or site-specific nucleases. Therefore, pluripotent stem cells can be employed to get insights into the role of c-CBL in (pluripotent) stem cells of primates. Based on the data presented in this study, we hypothesize that c-CBL has an important role in pluripotent primate stem cells of the marmoset. Furthermore, it is well known that c-CBL has an important role in the hematopoietic system. Taken together, the data support the view of an important function of c-CBL in primate stem cells. However, the experimental proof is still missing.

The amino acid sequence of the primate c-CBL proteins is highly conserved. This also suggests a highly conserved function of c-CBL in primates. In contrast, the sequences in non-primate species, including pig, dog, rat and mouse, exhibit significantly lower sequence conservation. These clear molecular differences in combination with physiological differences of the cells and tissues expressing c-CBL between primates and non-primate species (like the immune system, the testis and pluripotent stem cells) raise the question of whether the findings from mice can be transferred to primates. We believe that these differences support the view that separate analyses in primates or primate cells are required to understand the function of c-CBL in these species. In this context the isoform expression of c-CBL in the marmoset is also of interest. While the cultured stem cells, testis and skin fibroblast predominantly express the longer isoform containing the stretch of 10 AAs, the muscle, in particular, has an additional band of similar intensity to the larger band representing the smaller isoform. Interestingly, the stretch of histidines in the first exon of c-CBL is present in all species analyzed in this study. The additional form lacking these 10 AAs is only available for the marmoset in the ensemble database. Whether this transcript has any functional relevance remains for now unclear.

We had hypothesized that c-CBL might be a general stem cell marker. To test this hypothesis, we performed immunohistochemical analyses of different adult marmoset monkey tissues harboring adult stem cells including the endodermal epithelia of the gut (Takashima et al., 2013) and the stomach (Kim and Shivdasani, 2016) and the ectodermal epidermis. While the epidermis showed only very scattered, unclear and weak signals, the epithelia of the gut and the stomach were completely unstained, rejecting our hypothesis that c-CBL might be a general stem cell marker.

In summary, we confirm human c-CBL expression in hematopoietic cells and spermatogonia for the marmoset. We show, to our knowledge, for the first time that c-CBL is robustly expressed in undifferentiated primate pluripotent stem cells and is down-regulated during iPSC and ESC differentiation. However, c-CBL is not constitutively expressed in all adult stem cells; thus, c-CBL is not a general stem cell marker.

## Data Availability

Transcriptome data are available under GEO series accession numbers GSE64966 and GSE70897.
